# Sickle Trait Hemoglobin Does Not Influence *Anopheles* Biting Rates

**DOI:** 10.4269/ajtmh.25-0758

**Published:** 2026-04-07

**Authors:** Christine F. Markwalter, Emmah Kimachas, Erastus Kirwa, Joseph Kipkoech, Samuel Kahindi, Lucy Abel, Zay Yar Han, Judith N. Mangeni, Andrew A. Obala, Wendy Prudhomme O’Meara, Steve M. Taylor

**Affiliations:** ^1^ Duke Global Health Institute, Duke University, Durham, North Carolina, USA;; ^2^ Academic Model Providing Access to Healthcare, Eldoret, Kenya;; ^3^ School of Pure and Applied Sciences, Pwani University, Kilifi, Kenya;; ^4^ Division of Infectious Diseases, Duke University School of Medicine, Durham, North Carolina, USA;; ^5^ School of Public Health, College of Health Sciences, Moi University, Eldoret, Kenya;; ^6^ School of Medicine, College of Health Sciences, Moi University, Eldoret, Kenya

## Abstract

Children with sickle cell trait (HbAS) are protected against severe and symptomatic *Plasmodium falciparum* (*P. falciparum*) malaria. Although several within-host resistance mechanisms have been investigated, it is unknown whether this protection is attributable in part to reduced exposure to *P. falciparum* parasites via mosquito bites. In a 15-month cohort in Western Kenya, mosquito blood meals were matched to human hosts on the basis of short tandem repeat genotypes to determine individual mosquito biting rates. Using a multilevel multivariable model, mosquito biting behavior was assessed with respect to human β-globin genotypes, and no significant difference in biting rate was observed between individuals with wild-type (HbAA) and HbAS genotypes (biting rate ratio: 1.23; 95% CI: 0.86–1.77). These findings suggest that protection against malaria conferred by the sickle cell trait is unlikely to be attributable to reduced exposure to infectious mosquito bites.

## INTRODUCTION

Sickle cell trait (HbAS) protects individuals, particularly children, from severe and symptomatic malaria.^1^ Several mechanisms for HbAS-mediated protection have been proposed, including differentially acquired^2^ and innate immunity,^3^ enhanced parasite clearance,^4^ and reduced cytoadhesion.^5^^,^^6^ However, it is unknown whether this protection may be attributable in part to reductions in exposure to *Plasmodium falciparum* (*P. falciparum*) parasites.

Exposure to *Plasmodium* parasites is dependent on the frequency of mosquito–human interactions, which is influenced by host factors such as odor, body temperature, and carbon dioxide.^7^ Human blood characteristics may also influence attractiveness to mosquitoes. *Anopheles gambiae* (*An. gambiae*) fed more frequently on individuals in blood group O compared with other ABO types.^8^^,^^9^ In artificial feeding experiments, *Anopheles stephensi* preferred blood group AB, followed by A, B, and O.^10^ The β-globin variants hemoglobin S (HbS) and C have been associated with increased transmission of *P. falciparum* from humans to anopheline vectors;^11^ however, it is unclear if this association is due in part to increased contact with vectors. To date, no studies have been conducted to explore whether the biting preference of *Anopheles* mosquitoes is affected by the human host’s β-globin type.

The present study is designed to determine if β-globin genotype (HbAS versus wild-type HbAA) influences mosquito biting preferences. In a 15-month longitudinal cohort study in Western Kenya, 516 blood meals were matched to community members using short tandem repeat (STR) genotyping. The authors hypothesized that *Anopheles* biting rates may be associated with the sickle cell trait.

## MATERIALS AND METHODS

### Study population and sample collection.

The present analysis is a subset of a longitudinal, community-based cohort study of up to 75 households across five villages in Bungoma County in Western Kenya.^12^ For the parent cohort study, *Anopheles* mosquitoes were collected twice per month in the cohort households using mechanical aspiration. Female *Anopheles* mosquitoes were dissected into heads and thoraces, wings, and abdomens. Freshly blood-fed abdomens were pressed onto filter paper. Finger-prick dried blood spots (DBSs) were collected monthly from all household members. Bed net use and symptomatic malaria episodes were also recorded for all participants via monthly behavioral surveys and passive surveillance, respectively. Community members with symptoms were tested for malaria using rapid diagnostic tests (RDTs) and provided treatment with artemether–lumefantrine. From this cohort, the present analysis included people in four villages who consented to β-globin typing. Events were observed from July 2020 to September 2021, during which blood-fed mosquitoes were matched to hosts.

### Molecular methods.

#### Detection of P. falciparum via quantitative polymerase chain reaction testing.

This laboratory analysis has been published previously.^12^ Briefly, genomic DNA (gDNA) was extracted from human DBSs using Chelex-100 (Bio-Rad Laboratories, Inc., Hercules, CA) and measured in duplicate using a TaqMan real-time polymerase chain reaction (PCR) assay (Life Technologies Corporation, Austin, TX) targeting the *P. falciparum* *pfr364* motif, as previously described.^13^ Positivity was determined on the basis of cycle threshold values, and parasite densities were estimated using standard curves.

#### Human genotyping.

The process of genotyping via STR analysis has been published previously.^12^ Each participant’s DBS and every mosquito abdomen identified as blood-fed, for which a blood spot was present, were used for genotyping human blood using the Promega Geneprint 10 assay (Promega Corporation, Madison, WI).^14^ Each sample type was genotyped after gDNA was extracted using Chelex-100 and purified using Zymo RNA Clean & Concentrator (Zymo Research, Irvine, CA) until a volume of 10 *µ*L was reached.

#### Beta-globin genotyping.

Genomic DNA was extracted from 0.25″ DBS punches from each participant via Chelex-100 until a volume of 150 *µ*L was reached, and the DNA was genotyped for HbS using a TaqMan single nucleotide polymorphism (SNP) genotyping assay targeting the *rs334* variant that encodes HbS. TaqMan SNP genotyping assay C_1888768814_10 was used, and 1 *µ*L of gDNA template was amplified in a 5 *µ*L reaction using TaqPath ProAmp Master Mix, as directed. All reactions were performed in duplicate on a QuantStudio 5 machine (Applied Biosystems, Waltham, MA), using plates containing templates harboring known HbAA, HbAS, and HbSS as controls.

### Blood meal matching.

Blood meal matches to individuals who had consented to β-globin typing from Markwalter et al.^12^ were used in the present analysis. Short tandem repeat profiles of cohort members and mosquito blood meals were matched using the bistro R package (v 0.2.0),^14^ which enables matching for incomplete STR profiles and multisource blood meals. The number of monthly bites per participant was calculated as follows:

Monthly bites=b/(2t)×30 days/month,

where *b* is the total number of bites observed, and *t* is the time at risk (in months) based on the number of monthly surveys. Thus, *b/2t* is the estimated nightly biting rate, given that mosquito collections were performed twice monthly.

### Risk factor analysis.

A negative binomial regression was used to analyze the association between mosquito biting rates and β-globin genotypes (HbAA versus HbAS). The outcome was the number of mosquito bites matched to a person on a given night, and covariates included age, sex, the use of bed nets (determined using most recent monthly survey, with missing data filled in using the monthly survey after the bite), and *P. falciparum* infection status (determined via quantitative PCR testing at the nearest monthly sampling [within –30 to +7 days of the bite, unless an RDT yielded a positive result within –14 to 0 days of the bite, and then a negative result after treatment was administered]). The model included a random intercept at the person level and was adjusted for 1) transmission season, 2) the number of STR-typed mosquitoes in the household, 3) the number of household members, and 4) the number of people in the sleeping space.

For the *Anopheles* species-specific analyses, community members who slept in the household within 30 days of a household night and were STR-matched with at least one mosquito of that species were included. The outcome was the number of species-specific (*Anopheles funestus* [*An. funestus*] or *An. gambiae* s.s.) bites matched to a person on a given night, and the same covariates and adjustors listed above were included.

## STATISTICAL ANALYSES

All analyses and visualizations were performed in RStudio (v 2024.04.2 + 764)^15^ with R (v 4.3.1)^16^ using the following packages: tidyverse (v 2.0.0),^17^ ggpubr (v 0.6.0),^18^ glmmTMB (v 1.1.8),^19^ broom.mixed (v 0.2.9.4),^20^ DescTools (v0.99.50),^21^ and modelr (v 0.1.11).^22^ All code that supports analyses and figures is available on GitHub (Microsoft Corp., Redmond, WA): https://github.com/malaria-house/trait_biting.

## RESULTS

The analytic dataset consisted of 52 households distributed across four villages, followed from July 2020 to September 2021, in which 313 individuals with HbAA or HbAS genotypes were at risk of being bitten. The HbSS genotype was detected in one individual, who was excluded from analyses. In this context, 2,841 female *Anopheles* were collected, of which 1,491 were freshly fed. Of 890 blood meals that were STR-typed, 621 returned human alleles, and 516 were matched to individuals in the cohort, with a total of 545 biting events. A total of 3,677 DBSs were also collected, of which 910 (25%) tested positive for *P. falciparum*.

Among the 313 participants in this study, 244 (78%) had the HbAA genotype, and 69 (22%) had the HbAS genotype. There were no significant differences in sex or age distributions between the β-globin genotypes. Similarly, there were no statistical differences in the proportion of time infected with *P. falciparum* or the proportion of time participants slept under a net across β-globin types (Table 1).

**Table 1:** Characteristics of individuals with the HbAA and HbAS genotypes

Characteristic	HbAA (*N* = 244)	HbAS (*N* = 69)	*P*-Value*
Age, *n* (%)			
<5	32 (13)	6 (8.7)	0.5
5–15	86 (35)	28 (41)	
>15	126 (52)	35 (51)	
Sex, *n* (%)			
Female	124 (51)	34 (49)	0.8
Male	120 (49)	35 (51)	
Proportion of time^†^ participants slept under a net, median (Q1–Q3)	0.75 (0.33–1.00)	0.60 (0.27–1.00)	0.12
Proportion of time^†^ participants were infected with *P. falciparum*, median (Q1–Q3)	0.21 (0.14–0.33)	0.27 (0.14–0.38)	0.059

*P. falciparum* = *Plasmodium falciparum*; Q = quartile.

The individual with the HbSS genotype has been omitted from this table.

* Pearson’s χ^2^ test; Wilcoxon rank sum test.

^†^ Proportion of time based on monthly sampling.

**Figure 1.**
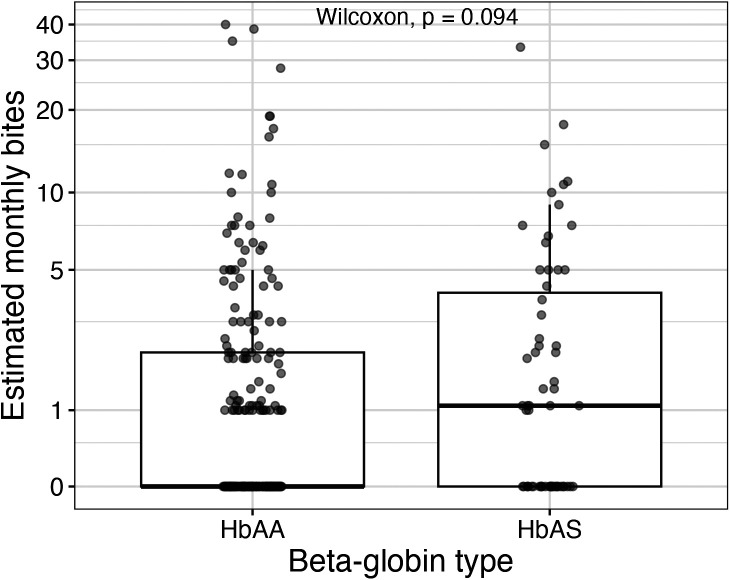
Estimated monthly bites per person by β-globin type. Each point represents one cohort member. Note that the *y*-axis is log-transformed to visualize biting rates between 0 and 10 bites per month.

The monthly biting rates for each individual were estimated from the observed number of bites and the time at risk over the 15-month study period. In both groups, biting was highly over-dispersed; the median number of estimated bites per month was zero for HbAA and one for HbAS, and the mean number of bites per month was 2.4 for HbAA (SD: 5.7) and 3.0 for HbAS (SD: 5.5; Figure 1).

**Figure 2.**
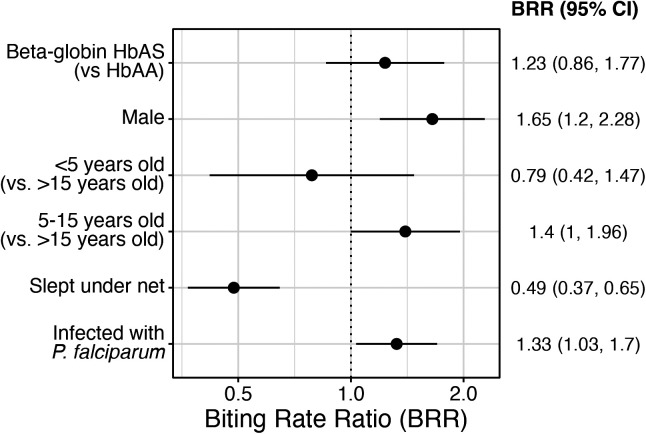
Risk factors for receiving female *Anopheles* bites. Biting rate ratios (points) and 95% CIs (bars) for covariates in risk factor analysis based on 1,325 person-nights.

In a multilevel multivariable model that included 1,325 person-nights at risk, participants with the HbAS genotype had similar mosquito biting rates (biting rate ratio [BRR]: 1.23; 95% CI: 0.86–1.77) compared with those with the HbAA genotype (Figure 2). Consistent with previous observations, higher biting rates were observed among males (versus females; BRR: 1.65; 95% CI: 1.20–2.28), children aged 5–15 years (versus adults; BRR: 1.40; 95% CI: 1.00–1.96), and participants with blood-stage infections (versus uninfected; BRR: 1.33; 95% CI:1.03–1.70). Lower biting rates were observed for people who reported sleeping under a net (BRR: 0.49; 95% CI: 0.37–0.65).

**Figure 3.**
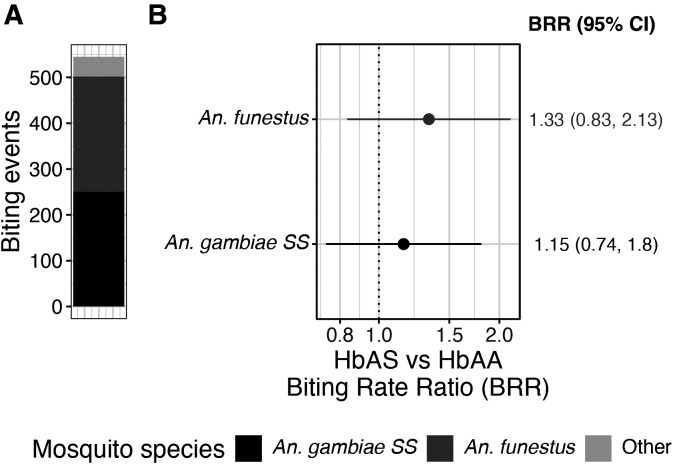
Biting rate ratios (HbAS versus HBAA) by *Anopheles* species. (**A**) Distribution of biting events by *Anopheles* species. (**B**) Biting rate ratios (points) 95% CIs (bars) for individuals with the HbAS genotype compared with those with the HbAA genotype for the two main species: *Anopheles funestus* and *Anopheles gambiae* s.s.

Next, the authors investigated whether the BRRs for people with HbAS vs HbAA genotypes differed across the two main vector species: *An. gambiae* s.s. and *An. funestus*. These two vectors together accounted for most (1,331/1491; 89%) of the blood-fed female *Anopheles* collected in study households. Among the 545 biting events observed, 250 (45.9%) involved *An. gambiae* s.s., and 252 (46.2%) involved *An. funestus* (Figure 3A). No difference was observed in the BRRs (HbAS versus HbAA) of species-specific models (Figure 3B); compared with participants with HbAA, community members with HbAS received bites at a similar rate from *An. funestus* (BRR: 1.33; 95% CI: 0.83–2.13) and *An. gambiae* s.s. (BRR: 1.15; 95% CI: 0.74–1.80).

**Figure 4.**
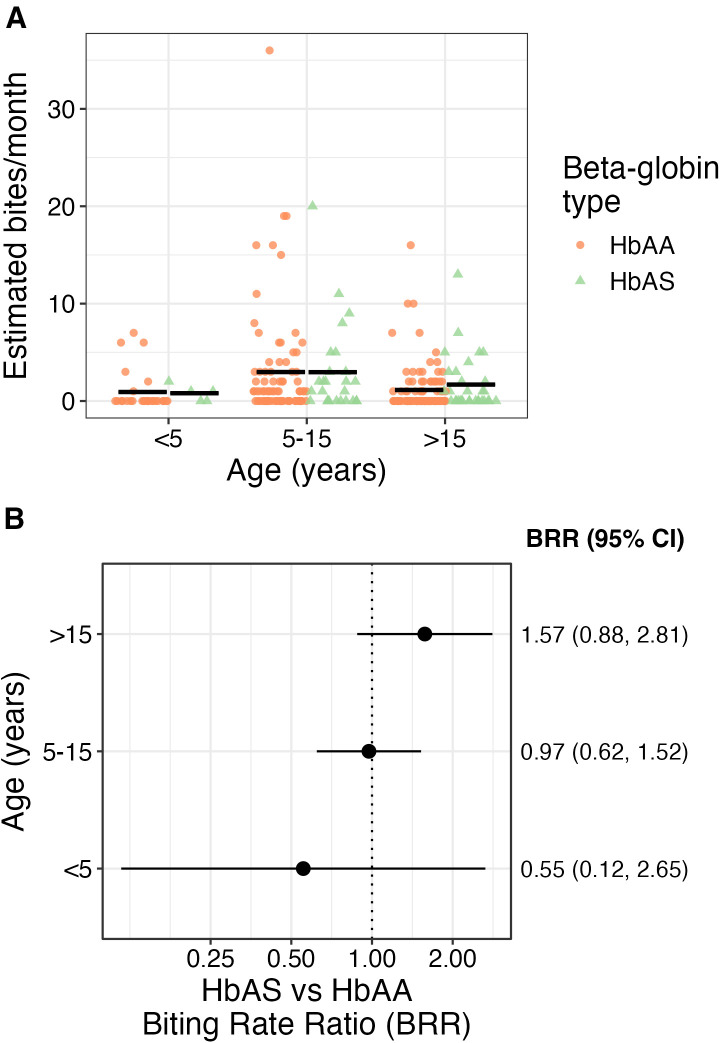
Female *Anopheles* biting rates by age group and β-globin type. (**A**) Individual estimated monthly bites received, stratified by age group and β-globin type (color). The black bar represents the mean monthly bites. (**B**) Biting rate ratios (points) and 95% CIs for individuals with the HbAS genotype compared with those with the HbAA genotype, stratified by age category.

Finally, the authors investigated whether age modified the effect of β-globin genotype on mosquito biting rates. The distribution of estimated mosquito bites per month stratified by age group (<5 years, 5–15 years, and >15 years) and β-globin genotype (AA versus AS; Figure 4A) reveals that the mean values (black bars) did not differ substantially between the two genotypes across any age category. Similarly, age-stratified models revealed no differences in BRRs for AS versus AA genotypes across age groups (Figure 4B); however, small sample sizes, particularly in the youngest age group, led to imprecise estimates.

## DISCUSSION

In the present analysis, the authors investigated whether sickle-trait hemoglobin influenced mosquito biting preference in a malaria-endemic setting in Western Kenya with a moderately high prevalence of HbAS (22%). Over 15 months across 52 households, human gDNA was detected in 621 blood-fed *Anopheles* mosquitoes. Genomic DNA found in 516 of these mosquitoes matched to cohort participants. No significant difference in biting rates was observed between participants with the HbAA genotype and those with the HbAS genotype, indicating that sickle-trait hemoglobin did not modify the risk of being bitten by anopheline mosquitoes. These observations were consistent between the two major vectors: *An. funestus* and *An. gambiae* s.s.

The HbAS genotype confers consistent protection against malaria via mechanisms that are incompletely understood. Therefore, the authors explored the possibility that protection could be mediated, in part, by simply reduced bites from malaria vectors. The premise that biting rates may differ between individuals with the HbAA and HbAS genotypes is based on previous reports of differential biting across blood group antigens.^8^^–^^10^^,^^23^ The mechanism of these differences is obscure, but the expression of ABO antigens on a wide variety of tissues, including endothelial and epithelial cells, renders these antigens more likely to be detectable to feeding mosquitoes. In contrast, nonerythroid expression of β-globin is rare^24^ and therefore may be less detectable to anopheline mosquitoes. Indeed, overall biting rates did not differ between people with the HbAA and HbAS genotypes, nor was reduced biting observed in age-specific groups, including children, who both have the highest incidence of malaria and benefit most from sickle-trait protection. Assuming that similar overall biting rates also reflect similar biting rates by infected mosquitoes, the study results indicate that protection is not mediated by reduced anopheline biting of people with the HbAS genotype.

The observation that people with the HbAS genotype do not experience enhanced biting is relevant to understanding how people with sickle-trait participate in onward transmission. The HbAS genotype does not protect against blood-stage parasitization, and although parasite densities are lower in symptomatic malaria cases among people with the HbAS genotype compared with those with the HbAA genotype,^25^ this difference has not been observed in asymptomatic infections,^25^^,^^26^ which comprise the vast majority of the infections for onward transmission in this cohort.^13^ Previous studies have revealed that individuals with the HbAS genotype infected with *P. falciparum* exhibit enhanced transmissibility^11^ compared with individuals with the HbAA genotype. Although this effect has primarily been attributed to elevated prevalences and densities of transmissible gametocytes^3^^,^^26^ and the subsequent increased likelihood of transmission to mosquitoes during feeding,^27^^,^^28^ this increased transmission could also be enhanced if the HbAS genotype increased human–vector contacts. Although people with the HbAS genotype exhibit higher gametocyte prevalences and densities, which may drive *Anopheles* host preference and increased biting rates as reported,^29^ the study results suggest that the HbAS genotype itself does not directly enhance vector biting.

The present study has limitations. The small number of children under 5 years of age in the study (*n* = 38) has limited the authors’ ability to precisely estimate BRRs specific to this age group, which suffers both the highest burden of malaria and the greatest protection from disease. Second, the captured mosquitoes represented only a small sample of all mosquitoes that fed within the community households. However, given the consistent collections, the study sample was likely not biased toward biting a specific group of participants. Finally, the approach of matching blood meals enables the successful measurement of mosquito feeds and does not capture mosquito bites without successful feeding, which may contribute to disease risk.

## CONCLUSION

The authors observed no difference in mosquito biting rates between people with the HbAS and HbAA genotypes across all age groups. Therefore, neither protection from malaria conferred by the sickle trait nor enhanced transmissibility of infections is likely attributable to differential exposure to mosquito bites.

## References

[1] Taylor SM Parobek CM Fairhurst RM, 2012. Haemoglobinopathies and the clinical epidemiology of malaria: A systematic review and meta-analysis. *Lancet Infect Dis* 12: 457–468. 22445352 10.1016/S1473-3099(12)70055-5 PMC3404513

[2] Williams TN Mwangi TW Roberts DJ Alexander ND Weatherall DJ Wambua S Kortok M Snow RW Marsh K, 2005. An immune basis for malaria protection by the sickle cell trait. PLoS Med 2: e128. 15916466 10.1371/journal.pmed.0020128 PMC1140945

[3] Gong L Maiteki-Sebuguzi C Rosenthal PJ Hubbard AE Drakeley CJ Dorsey G Greenhouse B, 2012. Evidence for both innate and acquired mechanisms of protection from *Plasmodium falciparum* in children with sickle cell trait. Blood 119: 3808–3814. 22327223 10.1182/blood-2011-08-371062 PMC3335384

[4] Ayi K Turrini F Piga A Arese P, 2004. Enhanced phagocytosis of ring-parasitized mutant erythrocytes: A common mechanism that may explain protection against falciparum malaria in sickle trait and beta-thalassemia trait. Blood 104: 3364–3371. 15280204 10.1182/blood-2003-11-3820

[5] Cholera R, , 2008. Impaired cytoadherence of *Plasmodium falciparum*-infected erythrocytes containing sickle hemoglobin. *Proc Natl Acad Sci USA* 105: 991–996. 18192399 10.1073/pnas.0711401105 PMC2242681

[6] Petersen JEV Saelens JW Freedman E Turner L Lavstsen T Fairhurst RM Diakité M Taylor SM, 2021. Sickle-trait hemoglobin reduces adhesion to both CD36 and EPCR by *Plasmodium falciparum*-infected erythrocytes. PLoS Pathog 17: e1009659. 34115805 10.1371/journal.ppat.1009659 PMC8221791

[7] Blanken SL Prudhomme O’Meara W Hol FJH Bousema T Markwalter CF, 2024. À la carte: How mosquitoes choose their blood meals. Trends Parasitol 40: 591–603. 38853076 10.1016/j.pt.2024.05.007 PMC11223952

[8] Wood CS Harrison GA Doré C Weiner JS, 1972. Selective feeding of *Anopheles gambiae* according to ABO blood group status. Nature 239: 165–165. 10.1038/239165a0 4561964

[9] Wood CS, 1974. Preferential feeding of *Anopheles gambiae* mosquitoes on human subjects of blood group O: A relationship between the ABO polymorphism and malaria vectors. Human Biol 46: 385–404. 4426600

[10] Anjomruz M, , 2014. Preferential feeding success of laboratory reared *Anopheles stephensi* mosquitoes according to ABO blood group status. Acta Trop 140: 118–123. 25151045 10.1016/j.actatropica.2014.08.012

[11] Gouagna LC Bancone G Yao F Yameogo B Dabiré KR Costantini C Simporé J Ouedraogo JB Modiano D, 2010. Genetic variation in human HBB is associated with *Plasmodium falciparum* transmission. Nat Genet 42: 328–331. 20305663 10.1038/ng.554

[12] Markwalter CF, , 2024. *Plasmodium falciparum* infection in humans and mosquitoes influence natural Anopheline biting behavior and transmission. Nat Commun 15: 4626. 38816383 10.1038/s41467-024-49080-9 PMC11139876

[13] Sumner KM Freedman E Abel L Obala A Pence BW Wesolowski A Meshnick SR Prudhomme-O’Meara W Taylor SM, 2021. Genotyping cognate *Plasmodium falciparum* in humans and mosquitoes to estimate onward transmission of asymptomatic infections. Nat Commun 12: 909. 33568678 10.1038/s41467-021-21269-2 PMC7875998

[14] Lapp Z Abel L Mangeni J Obala AA O’Meara WP Taylor SM Markwalter CF, 2023. bistro: An R package for vector bloodmeal identification by short tandem repeat overlap. Method Ecol Evol 15: 308–316. 10.1111/2041-210x.14277 PMC11218906 38962557

[15] Posit Team, 2023. RStudio: Integrated Development Environment for R. Boston, MA: Posit Software, PBC.

[16] R Core Team, 2023. R: A Language and Environment for Statistical Computing. Vienna, Austria: R Foundation for Statistical Computing.

[17] Wickham H, , 2019. Welcome to the Tidyverse. *JOSS* 4: 1686.

[18] Kassambara A, 2023. *ggpubr: “ggplot2” Based Publication Ready Plots*. Available at: https://cran.r-project.org/web/packages/ggpubr/index.html. Accessed March 18, 2026.

[19] Brooks ME Kristensen K Benthem KJ van, Magnusson A Berg CW Nielsen A Skaug HJ Mächler M Bolker BM, 2017. glmmTMB balances speed and flexibility among packages for zero-inflated generalized linear mixed modeling. *R J* 9: 378–400.

[20] Bolker B Robinson D, 2022. *broom.mixed: Tidying Methods for Mixed Models*. Available at: https://cran.r-project.org/web/packages/broom.mixed/broom.mixed.pdf. Accessed March 18, 2026.

[21] Signorell A, 2023. *DescTools: Tools for Descriptive Statistics*. Available at: https://andrisignorell.github.io/DescTools/. Accessed March 18, 2026.

[22] Wickham H, 2023. *modelr: Modelling Functions that Work with the Pipe*. Available at: https://cran.r-project.org/web/packages/modelr/modelr.pdf. Accessed March 18, 2026.

[23] Thornton C Doré CJ Willson JOC Hubbard JL, 1976. Effects of human blood group, sweating and other factors on individual host selection by species A of the *Anopheles gambiae* complex (Diptera, Culicidae). *Bull Entomol Res* 66: 651–663.

[24] Keller TCS Lechauve C Keller AS Brooks S Weiss MJ Columbus L Ackerman H Cortese-Krott MM Isakson BE, 2022. The role of globins in cardiovascular physiology. *Physiol Rev* 102: 859–892. 34486392 10.1152/physrev.00037.2020 PMC8799389

[25] Williams TN Mwangi TW Wambua S Alexander ND Kortok M Snow RW Marsh K, 2005. Sickle cell trait and the risk of *Plasmodium falciparum* malaria and other childhood diseases. J Infect Dis 192: 178–186. 15942909 10.1086/430744 PMC3545189

[26] Andolina C, , 2023. *Plasmodium falciparum* gametocyte carriage in longitudinally monitored incident infections is associated with duration of infection and human host factors. Sci Rep 13: 7072. 37127688 10.1038/s41598-023-33657-3 PMC10150352

[27] Robert V Tchuinkam T Mulder B Bodo JM Verhave JP Carnevale P Nagel RL, 1996. Effect of the sickle cell trait status of gametocyte carriers of *Plasmodium falciparum* on infectivity to anophelines. Am J Trop Med Hyg 54: 111–113. 8619431 10.4269/ajtmh.1996.54.111

[28] Ngou CM, , 2023. Influence of the sickle cell trait on *Plasmodium falciparum* infectivity from naturally infected gametocyte carriers. BMC Infect Dis 23: 317. 37165325 10.1186/s12879-023-08134-x PMC10173526

[29] Lacroix R Mukabana WR Gouagna LC Koella JC, 2005. Malaria infection increases attractiveness of humans to mosquitoes. PLoS Biol 3: e298. 16076240 10.1371/journal.pbio.0030298 PMC1182690

